# A Novel c-MET-Targeting Antibody-Drug Conjugate for Pancreatic Cancer

**DOI:** 10.3389/fonc.2021.634881

**Published:** 2021-03-17

**Authors:** Yangbing Jin, Zehui Zhang, Siyi Zou, Fanlu Li, Hao Chen, Chenghong Peng, Xiaxing Deng, Chenlei Wen, Baiyong Shen, Qian Zhan

**Affiliations:** ^1^Department of General Surgery, Pancreatic Disease Center, Research Institute of Pancreatic Diseases, Ruijin Hospital, Shanghai Jiao Tong University School of Medicine, Shanghai, China; ^2^State Key Laboratory of Oncogenes and Related Genes, Institute of Translational Medicine, Shanghai Jiao Tong University, Shanghai, China

**Keywords:** pancreatic ductal adenocarcinoma, SHR-A1403, c-Met antibody-drug conjugate, anti-tumour, cholesterol metabolism

## Abstract

Pancreatic ductal adenocarcinoma (PDAC) is the fourth leading cause of cancer-associated death in the United States and has a 5-year survival rate of <4%. Although much effort has been invested in the research and development of pancreatic cancer drugs over the past 30 years, due to the lack of effective targetable carcinogenic drivers, no new targeted therapies that can improve patient prognosis have been approved for clinical use. SHR-A1403 is a new c-mesenchymal-epithelial transition factor (c-MET) antibody-drug conjugate that can be used for the targeted treatment of PDAC with high c-MET expression. This study reports for the first time the application prospects of SHR-A1403 in preclinical models of PDAC. SHR-A1403 significantly inhibited the proliferation, migration, and invasion of pancreatic cancer cells and induced cell cycle arrest and apoptosis. These changes were caused by inhibition of intracellular cholesterol biosynthesis by SHR-A1403. Therefore, targeting c-MET through SHR-A1403 showed strong preclinical anti-tumour efficacy in pancreatic cancer. Our work suggests the potential application of c-MET-targeted antibody-drug conjugate treatment for PDAC in clinical practise.

## Introduction

Pancreatic ductal adenocarcinoma (PDAC) is the fourth leading cause of cancer-associated death in the United States and has a 5-year survival rate of <4% (1, 2). As one of the few malignancies whose mortality is almost equal to its incidence, its dismal prognosis is mainly due to the highly aggressive and early metastatic nature of PDAC. Most patients present with extra-pancreatic dissemination at diagnosis (3). Furthermore, the biological barrier and immune microenvironment created by the proliferative matrix create unfavourable conditions for drug delivery, and the effects of chemotherapy and targeted therapy are still very limited. Although much effort has been invested in the development of pancreatic cancer drugs over the past 30 years, due to the lack of effective targetable carcinogenic drivers, no new drugs that can improve patient prognosis have been developed (4, 5). New treatment strategies are urgently required.

c-mesenchymal-epithelial transition factor (c-MET), also known as hepatocyte growth factor receptor, is a receptor tyrosine kinase encoded by the proto-oncogene *MET*. It is currently the only known receptor with high affinity for its naturally occurring ligand, hepatocyte growth factor (HGF). This pathway regulates a variety of important cellular responses, including proliferation, apoptosis, motility, and morphogenesis (6–9). Under normal circumstances, activation of the c-MET pathway is regulated to maintain homeostasis, but during tumorigenesis, c-MET signalling becomes dysregulated. Dysregulation of this pathway can occur through mechanisms including c-MET overexpression, genomic amplification, mutation, or alternative splicing (10). In pancreatic cancer, increased transcription leads to overexpression of c-MET, which is one of the most common causes of constitutive c-MET activation, and this increased c-MET promotes tumour occurrence and development through multiple mechanisms (7). In the past decade, treatments for abnormal HGF/MET signalling have been rapidly developed, including biological drugs called antibody-drug conjugates (ADCs), which combine MET-based targeted therapy and immune therapy. By targeting their cytotoxic payload to the tumour site through the antibody component, ADCs can improve the effect of chemotherapy and reduce systemic exposure and toxicity (11, 12). The expression of c-MET in pancreatic tumour tissues is 5-7-fold higher than that of adjacent tissues, which creates a large therapeutic safety window for ADCs targeting c-MET (13, 14).

SHR-A1403 is a novel c-MET ADC composed of a humanised anti-c-MET IgG2 monoclonal antibody (mAb) covalently linked to a proprietary uncleavable thioether linker and the new cytotoxic small-molecule drug SHR152852. The drug has completed preclinical pharmacokinetic studies in rodents and non-human primates, which showed that SHR-A1403 has a high affinity for human c-MET. Furthermore, almost no free toxin was detected in rats and cynomolgus monkeys after administration (15). SHR-A1403 has shown significant anti-tumour activity in a variety of tumour cell lines with high c-MET levels, xenograft mouse models, and patient-derived xenografts (PDX) models of HCC. Based on these results and those of previous studies, it can be speculated that the toxin released by internalisation and translocation to the lysosome is the main mechanism of SHR-A1403's anti-tumour activity (16).

In the present study, we explored the anti-tumour efficacy and underlying mechanism of SHR-A1403 in *in vitro* and *in vivo* models of PDAC to determine whether it can be a novel treatment for pancreatic cancer. This is the first in-depth study of SHR-A1403 in a preclinical model of pancreatic cancer, which is of great significance.

## Materials and Methods

### Drugs and Reagents

SHR-A1403 (ADC), SHR-A1403 mAb (the naked anti-c-MET monoclonal antibody), and the free toxin SHR152852 were provided by Shanghai Hengrui Pharmaceutical Co., Ltd., China. c-MET (ab216574), HMGCR (ab174830), LSS (ab124785), DHCR24 (ab181062), goat anti-rabbit IgG (ab6721), and goat anti-mouse IgG (ab6789) antibodies were purchased from Abcam, and GAPDH (2118L), SNAIL (3879S), and β-catenin (8480P) antibodies were purchased from Cell Signalling Technology. SREBP2 (28212-1-AP), E-cadherin (20874-1-AP), N-cadherin (66219-1-Ig), and vimentin (10366-1-AP) antibodies were purchased from Proteintech. The Cholesterol Quantitation Kit (MAK043-1KT) and IGEPAL(R) CA-630 (I8896-50ML) were purchased from Sigma-Aldrich, and the Cholesterol Assay Kit (Cell-Based) (ab133116) was purchased from Abcam.

### Samples and Immunohistochemistry (IHC)

The specimens used in this experiment were all provided by the Department of Pancreatic Surgery, Ruijin Hospital, Shanghai Jiaotong University School of Medicine, from January 2014 to December 2018. All patients were at least 18 years old and were pathologically confirmed as cases of pancreatic ductal adenocarcinoma following diagnosis and surgical treatment in this hospital. The exclusion criteria were as follows: malignant tumours that occurred in other organs at the same time, previous systemic treatment, incomplete clinical data, and loss to follow-up. The research protocol was approved by the ethics committee of Shanghai Ruijin Hospital (No. 17, 2019). Relevant experiments involving human specimens were conducted in strict accordance with the Human Experiment Ethics Committee and the Declaration of Helsinki, 2013 revision. Tissue samples from 69 patients with pancreatic cancer were collected, and the clinicopathological data of the patients including name, sex, age, tumour size, TNM stage, nerve invasion, lymph node metastasis, distant metastasis, and follow-up data were recorded in detail until the final event of the patient occurred. The clinicopathological characteristics of these patients are presented in Table 1. All slides were sent to Wuhan Servicebio Co., Ltd. for IHC. The 5-μm tissue samples were treated with 4% paraformaldehyde to fix the paraffin-embedded sections, and the primary and secondary antibodies were diluted according to the manufacturer's instructions to achieve the best dilution (1:500, rabbit anti-c-MET antibody, ab216574; 1:1,000, goat anti-rabbit IgG, ab6721). The samples were developed with a chromogenic substrate for about 10 min at room temperature. Brown-yellow colour of the cytoplasm or the cell membrane was considered a positive staining. The scoring formula was ∑pi (p1 × i1+p2 × i2...) where i represents the staining intensity of a cell and p the percentage of the total cell area occupied by the same staining intensity. First, we determined the proportion of positive cells as 0 (0–25%), 1 (26–50%), 2 (51–75%), or 3 (76–100%) points. The staining intensity ranging from 0 to 3 points was categorised as no, weak, medium, and strong staining, respectively. The sum of these two-point systems determined the final tissue section score. A total score >7 was considered a high c-MET expression, otherwise, it was considered a low expression.

**Table 1:** Correlation between c-MET expression and clinicopathological features of patients with pancreatic cancer.

**Characteristics**		***n* = 69**	**c-MET**		***P*-value**
			**Low expression**	**High expression**	
Age	≤65	45 (65.2%)	21(72.4%)	24 (60.0%)	0.317
	>65	24 (34.8%)	8 (27.6%)	16 (40.0%)	
Sex	Male	41 (59.4%)	17 (58.6%)	24 (60.0%)	>0.999
	Female	28 (40.6%)	12 (41.4%)	16 (40.0%)	
Tumour size	≤4	54 (78.3%)	24 (82.8%)	30 (75.0%)	0.559
	>4	15 (21.7%)	5 (17.2%)	10 (25.0%)	
Pathologic stage	I+II	41 (59.4%)	19 (65.5%)	22 (55.0%)	0.460
	III+IV	28 (40.6%)	10 (34.5%)	18 (45.0%)	
T stage	T1+T2	43 (62.3%)	20 (69.0%)	23 (57.5%)	0.451
	T3+T4	26 (37.7%)	9 (31.0%)	17 (42.5%)	
N stage	N0	20 (29.0%)	8 (27.6%)	12 (30.0%)	0.984
	N1	36 (52.2%)	16 (55.2%)	20 (50.0%)	
	N2	13 (18.8%)	5 (17.2%)	8 (20.0%)	
M stage	M0	65 (94.2%)	29 (100.0%)	36 (90.0%)	0.133
	M1	4 (5.8%)	0 (0.0%)	4 (10.0%)	
Perineural invasion	Negative	4 (5.8%)	4 (13.8%)	0 (0.0%)	0.028
	Positive	65 (94.2%)	25 (86.2%)	40 (100.0%)	

### Cell Culture

Six cell lines, including the normal human pancreatic ductal cell line HPNE and five pancreatic cancer cell lines (Aspc-1, BxPC-3, MiaPaCa-2, PanC-1, and Patu-8988) were obtained from the cell bank of the Chinese Academy of Sciences. Aspc-1 cells were cultured in RPMI-1640 medium supplemented with 10% foetal bovine serum, 100 U/mL penicillin, and 100 U/mL streptomycin at 37°C in a humidified 5% CO_2_ atmosphere. Other cell lines were cultured in DMEM under the same conditions.

### Western Blotting

After treatment, cells were lysed with RIPA buffer (R0278; Sigma-Aldrich) to extract protein. The PAGE Gel Quick Preparation kit (10%) (PG112; EpiZyme) was used to configure SDS-PAGE gel. The same amount of protein sample was added to each lane with a specific protein-loading pipette tip, as well as 5 μl of protein molecular weight standard reference (marker) to detect the protein band position. The protein was transferred to a PVDF membrane. After blocking with 5% skim milk, the membrane was incubated with the primary antibody at 4°C overnight and subsequently with the corresponding secondary antibody for 2 h at room temperature. The target protein component separated by electrophoresis was detected by autoradiography. The primary and secondary antibodies used for western blotting are listed in section drugs and reagents.

### RNA Extraction and Quantitative Reverse Transcription Polymerase Chain Reaction (qRT-PCR)

Trizol and related reagents were used to extract cellular RNA, which was reverse-transcribed into cDNA. SYBR Green fluorescent dye was added to the qRT-PCR reaction to ensure that the increase in fluorescence signal was completely synchronised with the increase in PCR products. Ct values were obtained through real-time detection of the corresponding fluorescence signal intensity. Several standard samples with known template concentrations were used as controls to obtain the copy number of the target gene in the test specimen. PCR primers (Supplementary Table 1) were designed and synthesised by Shanghai Sangon Biotech.

### Cell Proliferation Assay

Cells were seeded into 96-well plates at a density of 5,000 cells per well and cultured for 24 h. Then, cells were incubated with different concentrations of SHR-A1403 for 72 h, at which point 100 μL of complete culture medium containing 10 μL CCK-8 reagent was added to each well. The mixture was incubated at 37°C in an incubator for 2 h. A microplate reader (Epoch; BioTek, Winooski, VT, USA) was used to measure the absorbance at 450 nm. At least three independent experiments were repeated for each condition.

### Colony Formation Assay

The cells were seeded into 6-well plates at a density of 2,000 cells per well and cultured for 24 h, at which point they were treated with different concentrations of SHR-A1403. After 2 weeks, cells were stained with 0.1% crystal violet solution, and the number of colonies was counted to assess the degree of cell proliferation.

### Transwell Assay

Cells were cultured in SHR-A1403-containing medium for 72 h and then seeded into the upper chamber of a transwell plate containing 200 μL of serum-free medium at a cell density of 100,000 per well. Complete medium containing 10% foetal bovine serum was added to the lower chamber. Cells were incubated in a 5% CO_2_ incubator at 37°C for 48 h and then stained with crystal violet. Five areas were randomly selected under the microscope for cell counting and statistical analysis.

### Cell Cycle Analysis

Cells were seeded into 6-well plates and cultured in serum-free medium for 24 h, at which point different concentrations of SHR-A1403 were added. After 72 h, the cells were collected and fixed in 75% ethanol at −20°C overnight. Subsequently, the DNA was stained with propidium iodide (PI) for 30 min at 37°C in the dark. A FACSCalibur flow cytometer was used to quantify the intracellular DNA content in 5 × 10^5^ cells. ModFit Lt Mac V3.0 software was used to analyse the data.

### Apoptosis Assay

The cells were seeded into 6-well plates and treated under different conditions for 72 h. The cells were then trypsinised and stained with 5 μL of Annexin V-FITC and 5 μL of PI and analysed by flow cytometry (Becton-Dickinson, Franklin Lakes, NJ, USA). Three independent experiments were performed.

### Lentiviral Transduction

Lentivirus containing c-MET shRNA was constructed to test the off-target effects of SHR-A1403. The transfer plasmid was transfected using riboFECT™ CP Reagent according to the manufacturer's instructions (Bioegene, Shanghai, China). Aspc-1 cells were transduced with the c-MET shRNA lentivirus.

### Next-Generation Sequencing and Ingenuity Pathway Analysis (IPA)

Aspc-1 cells were treated with dimethyl sulfoxide (control) or SHR-A1403 (200 ng/mL) for 72 h. Standard extraction methods were used to extract RNA from the cells, followed by strict quality control of the RNA samples. The samples were then sent to Shanghai Genechem Co., Ltd. for RNA sequencing (RNAseq). This next-generation sequencing analysis uses quantitative nuclease protection to examine 2,560 markers related to tumour biology. Ribosomal RNA was removed from total RNA to obtain mRNA. Subsequently, the mRNA was randomly interrupted with divalent cations in NEB Fragmentation Buffer, and the library was constructed according to the NEB library construction method. After the library quality was confirmed, the different libraries were pooled according to the effective concentration and target offline data volume requirements for Illumina sequencing. The screening criteria for RNAseq were set to padj < 0.05 and |log2FoldChange| > 0.0.

Pathway analysis was carried out to determine the functional significance of differentially expressed genes after SHR-A1403 treatment. Genes with ≥ 1.5-fold changes compared to the control cells were entered into QIAGEN's IPA for analysis of involved pathways. The program was used with its default settings. According to the IPA standard, a Z-score ≥ 2 means that a pathway is significantly activated, and a Z-score ≤ −2 means that a pathway is significantly inhibited. We combined the activation Z-score algorithm, *P*-value, and ratio to predict the degree of activation or inhibition of any given pathway and the number of differentially expressed genes involved in the pathway.

All raw data related to sequencing have been uploaded to NCBI BioProject (ID: PRJNA681694).

### Semi-Quantitative Cholesterol Fluorescence Assay

Cells were plated into a 96-well plate at 3 × 10^4^ cells per well and treated for 72 h. Histochemical staining was performed, and U18666A (a cholesterol transport inhibitor) was used as a positive control. Fluorescence was assessed using a fluorescence microscope (λ_ex_ = 340–380 nm/λ_em_ = 385–470 nm). Pictures were immediately taken, and experimental data were analysed.

### Quantitative Cholesterol Detection

Aspc-1 cells were plated into 6-well plates and treated with different concentrations of SHR-A1403 for 72 h. A cholesterol quantification kit was used to determine the concentration of free cholesterol and cholesteryl esters. The total cholesterol concentration was determined by a coupled enzyme assay, which results in a colorimetric (570 nm)/fluorometric (λ_ex_ = 535 nm/λ_em_ = 587 nm) product proportional to the cholesterol present.

### *In vivo* Study

Female BALB/c nude mice (4–6 weeks old) were purchased from Shanghai PHENOTEK Biotechnology Co., Ltd. Aspc-1 cells were injected subcutaneously to create xenotransplantation models. When the average tumour volume reached ~100–200 mm^3^, the tumour-bearing mice were randomly divided into groups and intravenously injected with sterile injection, SHR-A1403 mAb, SHR152852, SHR-A1403 alone, or a combination of SHR-A1403 mAb and SHR152852. The tumour volume was calculated as (length × width^2^)/2, and weight was monitored as an indicator of overall health. All animal experiments were conducted in accordance with the guidelines of the Institutional Animal Care and Use Committee of the Shanghai Institute of Materia Medica, Chinese Academy of Sciences.

### Data Analysis

All data were analysed using GraphPad Prism 7 software (GraphPad Software, Inc.). Non-linear regression analysis was performed to generate a dose-response curve and calculate the IC_50_ value. The independent samples *t*-test was used to analyse differences between two groups. Comparison among multiple groups was performed using one-way analysis of variance. Pearson's χ^2^ test and Fisher's exact test were used to analyse the correlation between c-MET expression and clinicopathological characteristics. The Kaplan-Meier method was used to draw the survival curve, and the difference between two curves was analysed using the log-rank test. The results of repeated experiments are expressed as the mean ± standard deviation. A *P*-value of <0.05 was defined as statistically significant.

## Results

### c-MET Is Upregulated in PDAC Tissues and Associated With Poor Prognosis

**Figure 1:**
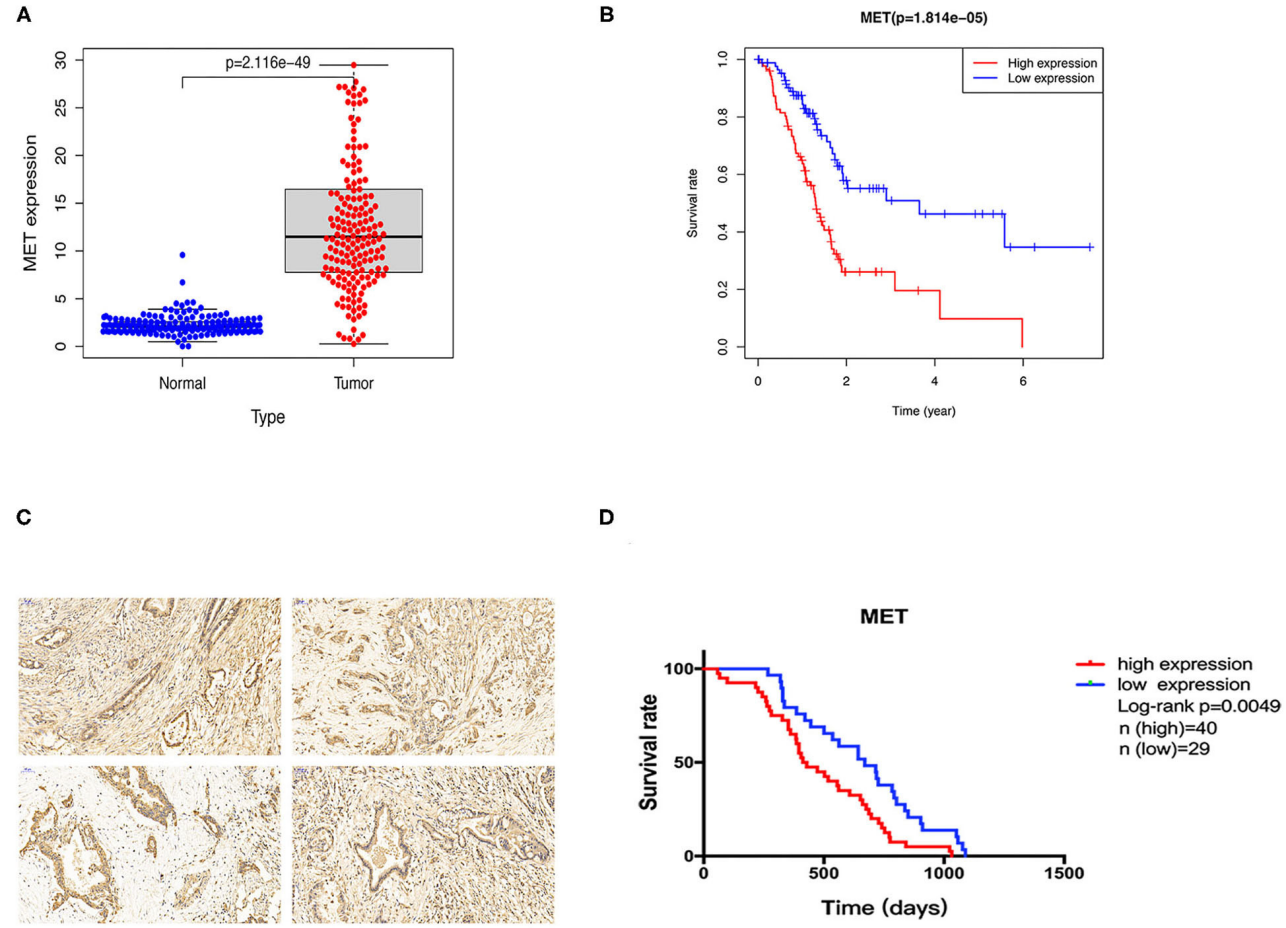
Expression and prognostic potential of c-MET in pancreatic cancer. **(A)** The TCGA and GTEX databases were used to compare the relative *MET* expression levels in 182 pancreatic cancer tumour tissues and 171 normal tissues. **(B)** A Kaplan-Meier curve was drawn using patient survival data from the TCGA database. **(C)** Sixty-nine pancreatic cancer tumour tissues collected from patients at our centre were used for c-MET IHC. **(D)** Kaplan-Meier survival analysis using patient follow-up data from our centre. *P* < 0.05 was considered statistically significant. c-MET, c-mesenchymal-epithelial transition factor; TCGA, The Cancer Genome Atlas; GTEX, The Genotype-Tissue Expression; IHC, immunohistochemistry.

To explore the *MET* expression pattern in PDAC and its relationship with prognosis, we compared the difference in *MET* expression between cancer tissue and healthy pancreatic tissue using the transcriptome data obtained from the public databases The Cancer Genome Atlas (TCGA) and The Genotype-Tissue Expression (GTEX). After screening by relevant conditions, the TCGA database included 182 PDAC samples and four normal samples, and the GTEX database included 167 normal samples. The results showed that the expression of *MET* in the tumour tissues of PDAC patients was significantly higher than that in the normal tissues (Figure 1A), and the Kaplan-Meier analysis showed that high *MET* expression was correlated with a significant reduction in overall survival (Figure 1B). To determine the expression level of c-MET in PDAC patients in our centre and its relationship with prognosis, we first performed IHC staining on 69 PDAC clinical specimens obtained in our hospital to analyse c-MET expression in paraffin-embedded specimens. As shown in Figure 1C, c-MET expression was significantly upregulated in PDAC specimens. The IHC results were scored according to the staining intensity and per cent of tumour cells stained, and clinical samples were classified as having high or low c-MET expression. The Kaplan-Meier analysis was then used to compare survival between these two groups. Patients with high c-MET expression had a significantly reduced overall survival rate (Figure 1D). Pearson's χ^2^ test and Fisher's exact test were used to classify and analyse the clinicopathological data of these 69 patients. Patients with high expression of c-MET were more likely to have a significantly increased neurological invasion, and they were also more prone to metastasis (Table 1).

### SHR-A1403 Potently Inhibits the Proliferation of PDAC Cells With High c-MET Expression

**Figure 2:**
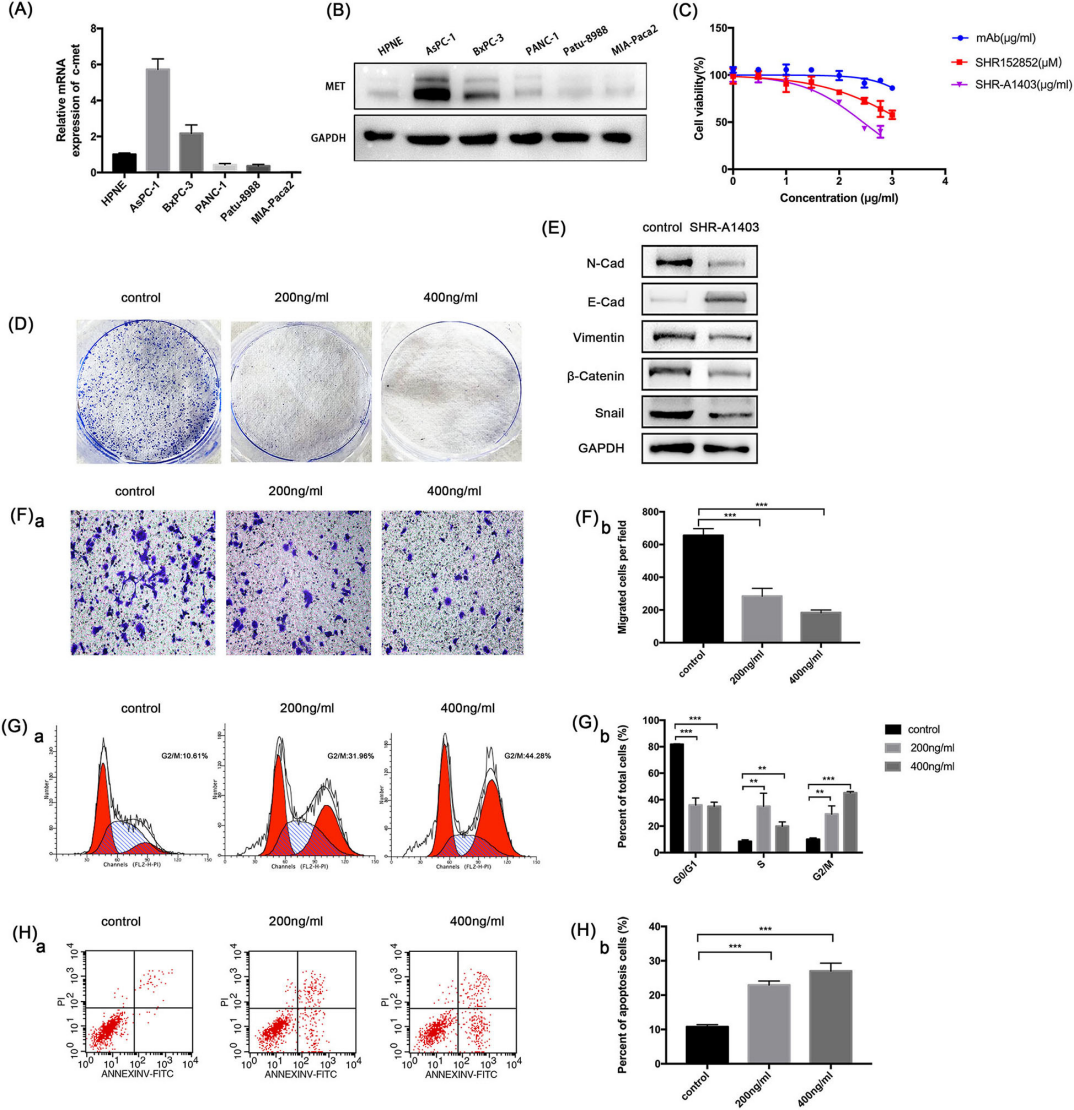
*In vitro* anti-tumour activity of SHR-a14A1403 in PDAC cells. **(A)** qRT-PCR analysis was used to detect relative *MET* expression in a normal pancreatic ductal cell line (HPNE) and five pancreatic cancer cell lines. **(B)** Western blot analysis was used to detect the expression of c-MET in a normal pancreatic ductal cell line (HPNE) and five pancreatic cancer cell lines. **(C)** IC_50_ values were determined using CCK-8 assays by treating cells with different concentrations of SHR-A1403 for 72 h. Data are presented as means ± *SD* of three independent experiments. **(D)** Colony formation assay to detect the proliferation of Aspc-1 cells treated with different concentrations of SHR-A1403. **(E)** Expression of EMT-related proteins after SHR-A1403 treatment. **(F)** Transwell assay to detect the migration ability of Aspc-1 cells treated with different concentrations of SHR-A1403. Migrated cells were quantified using ImageJ. **(G)** Cell cycle analysis to detect cell cycle changes in Aspc-1 cells treated with different concentrations of SHR-A1403. GraphPad Prism7 was used for quantitative statistics. **(H)** Apoptosis assay in Aspc-1 cells treated with different concentrations of SHR-A1403. Graphpad Prism 7 was used for quantitative statistics. **P < 0.01, ***P < 0.001. PDAC, pancreatic ductal adenocarcinoma; qRT-PCR, quantitative reverse transcription polymerase chain reaction; SD, standard deviation; EMT, epithelial-mesenchymal transition.

To understand the role of c-MET in PDAC, we used qRT-PCR and western blotting to evaluate the c-MET expression in a normal pancreatic duct epithelial cell line (HPNE) and five pancreatic cancer cell lines. The c-MET expression levels in pancreatic cancer cell lines were significantly increased compared to the expression in HPNE cells, particularly in Aspc-1 cells (Figures 2A,B). Therefore, we used Aspc-1 cells in subsequent experiments to evaluate the effects of SHR-A1403. We first compared the effects of SHR-A1403 with those of SHR-A1403 mAb and the free toxin SHR152852. SHR-A1403 potently inhibited proliferation of Aspc-1 cells, with an IC_50_ value of 281.3 ng/mL (Figure 2C). However, SHR152852 only weakly inhibited Aspc-1 cell proliferation, with an IC_50_ value of 1,749 nM, and the effect of SHR-A1403 mAb on the proliferation of Aspc-1 cells was almost negligible, with an IC_50_ of 5,435 ng/mL. Therefore, SHR-A1403 had a higher potency than SHR-A1403 mAb. Colony formation assays further illustrated the inhibitory effect of SHR-A1403 on cell proliferation (Figure 2D).

### SHR-A1403 Regulates Epithelial-Mesenchymal Transition (EMT) and the Migratory Ability of PDAC Cells

EMT is an important event for tumour metastasis because it makes cancer cells acquire aggressive behaviours and develop metastatic growth characteristics. Therefore, we hypothesised that SHR-A1403 may inhibit EMT in PDAC cells. To test this, we analysed the expression of EMT-related proteins. Aspc-1 cells were treated with SHR-A1403 or vehicle for 72 h and then subjected to western blot analysis. The results showed that the expression of N-cadherin, the mesenchymal marker vimentin, and the zinc finger transcription factor Snail were significantly increased in the cells treated with SHR-A1403, whereas the expression of the epithelial marker E-cadherin was decreased (Figure 2E). These results indicate that SHR-A1403 may inhibit EMT in PDAC cells.

To determine the effect of SHR-A1403 on the migration ability of PDAC cells, we conducted a transwell migration assay. Aspc-1 cells were pretreated with different concentrations of SHR-A1403 and placed into the upper chamber, and we counted the number of cells that migrated through the chamber after 48 h. The results showed that SHR-A1403 inhibited cell migration, and the inhibitory effect was increased with an increase in concentration (Figure 2F). Therefore, we confirmed that SHR-A1403 has a concentration-dependent inhibitory effect on migration of PDAC cells.

### SHR-A1403 Promotes G2/M Cell Cycle Arrest and Increases Apoptosis in PDAC Cells

To identify whether SHR-A1403 can alter cell cycle progression, Aspc-1 cells were incubated with serum-free culture medium for 24 h to synchronise the cell cycle and then treated with different concentrations of SHR-A1403 for 72 h. Cells were then stained with PI and analysed by flow cytometry. Compared to control cells, cells treated with SHR-A1403 exhibited G2/M phase arrest. Additionally, the accumulation of cells in the G2/M phase became more pronounced with increased SHR-A1403 concentrations (Figure 2G). Next, we analysed apoptosis by flow cytometry using FITC and PI staining. The percentage of apoptotic cells was significantly higher in SHR-A1403-treated cells than in control cells (Figure 2H). Therefore, our results indicate that SHR-A1403 can block cells in the G2/M phase and induce apoptosis.

### Next-Generation Sequencing Analysis of Cells Treated With SHR-A1403

**Figure 3:**
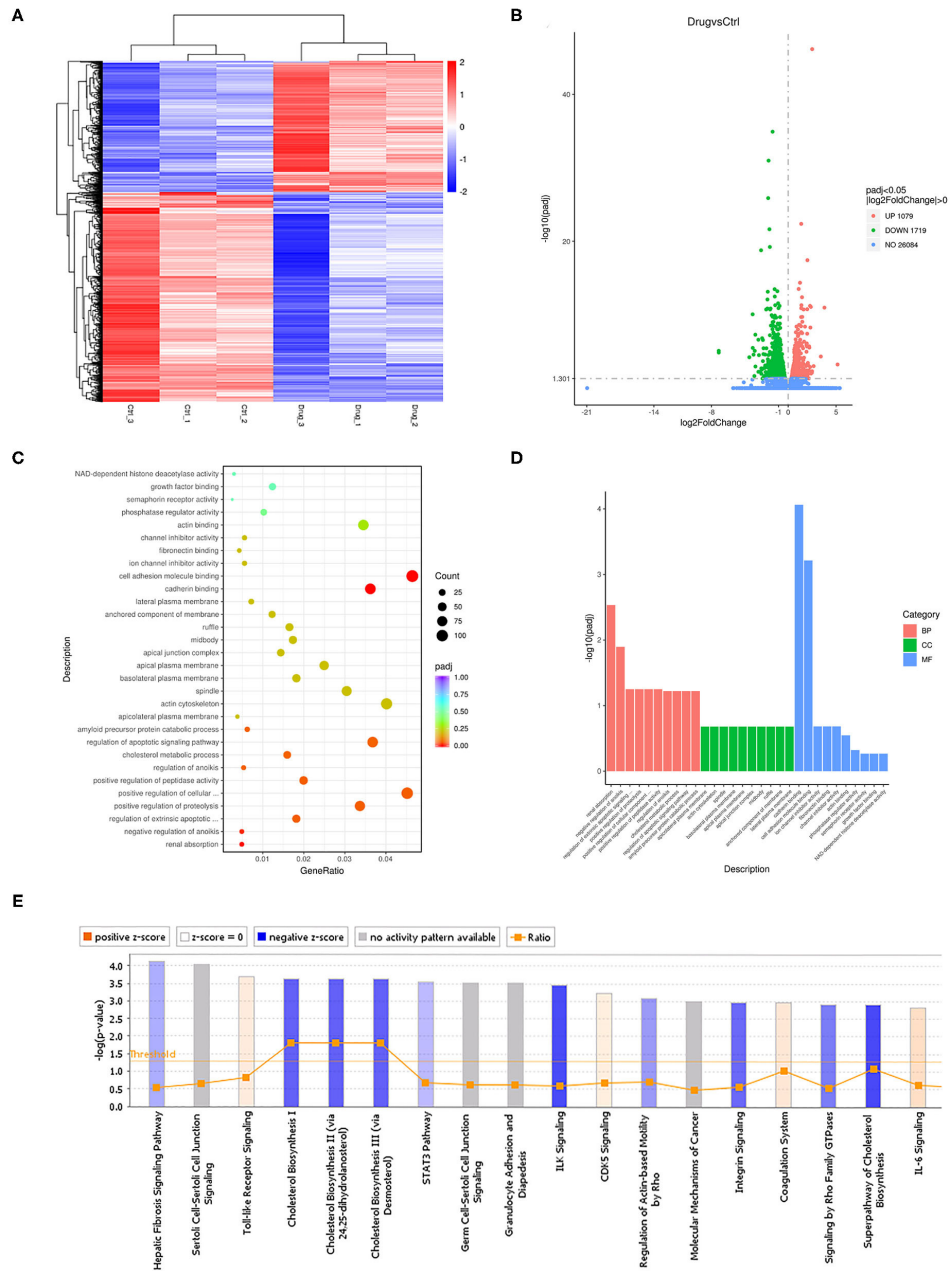
Cellular mechanisms underlying the anti-tumour effect of SHR-A1403. **(A)** Heatmap showing the upregulated and downregulated genes in cells treated with SHR-A1403 compared to control-treated cells. **(B)** Volcano plot showing the upregulated and downregulated genes in cells treated with SHR-A1403 compared to control-treated cells. **(C,D)** Pathway enrichment analysis showed that differentially expressed genes were enriched in pathways involved in migration, apoptosis, and cholesterol metabolism. **(E)** IPA showed that the cholesterol biosynthesis pathway was significantly inhibited by SHR-A1403. IPA, ingenuity pathway analysis.

To study the mechanism by which SHR-A1403 affects the biological function of PDAC cells, we performed RNAseq on Aspc-1 cells treated with 200 ng/mL SHR-A1403 and control cells. A total of 2,798 differentially expressed genes were detected, of which 1,079 were upregulated and 1,719 were downregulated. The heatmap and the volcano plot displaying the distribution of differential genes are shown in Figures 3A,B, respectively. Subsequent gene set enrichment analyses of differentially expressed genes showed that genes involved in cell migration, apoptosis, and cholesterol metabolism were all enriched at high levels (Figures 3C,D). IPA was used to adjust the sequencing threshold to FDR < 0.05 and |FC| > 1.5 and included all differentially expressed genes. We identified 96 IPA canonical pathways that were significantly involved (*P* < 0.05). Figure 3E lists the 18 most significantly affected pathways. This figure visually shows the trend of the expression of each gene in the pathway in the experimental results, which provides a basis for the study of the molecular mechanisms involved in changes between sample groups. Genes involved in cholesterol biosynthesis were significantly inhibited by treatment with SHR-A1403.

SHR-A1403 is an antibody-conjugated targeted drug. To determine whether the drug has off-target effects, we constructed a c-MET knockdown (KD) Aspc-1 cell line using lentiviral transduction. KD cells were then treated with SHR-A1403 or vehicle for 48 h, and then RNA was extracted for RNAseq analysis. A total of 10,065 differential genes were obtained, of which 5,029 were upregulated and 5,036 were downregulated. The heatmap and volcano plot that visually display the distribution of differentially expressed genes are shown in Supplementary Figures 1A,B, respectively. The differentially expressed genes and pathways obtained from RNAseq of wild-type cells were compared with those obtained from RNAseq of KD cells (Supplementary Figures 1C,D). GO enrichment analysis results of the KD control cells and SHR-A1403-treated cells indicated that there was a clear difference from the results of the wild-type cell enrichment analysis, indicating that this was not an off-target effect (Supplementary Figures 1E,F).

### SHR-A1403 Inhibits Cholesterol Biosynthesis in PDAC Cells

**Figure 4:**
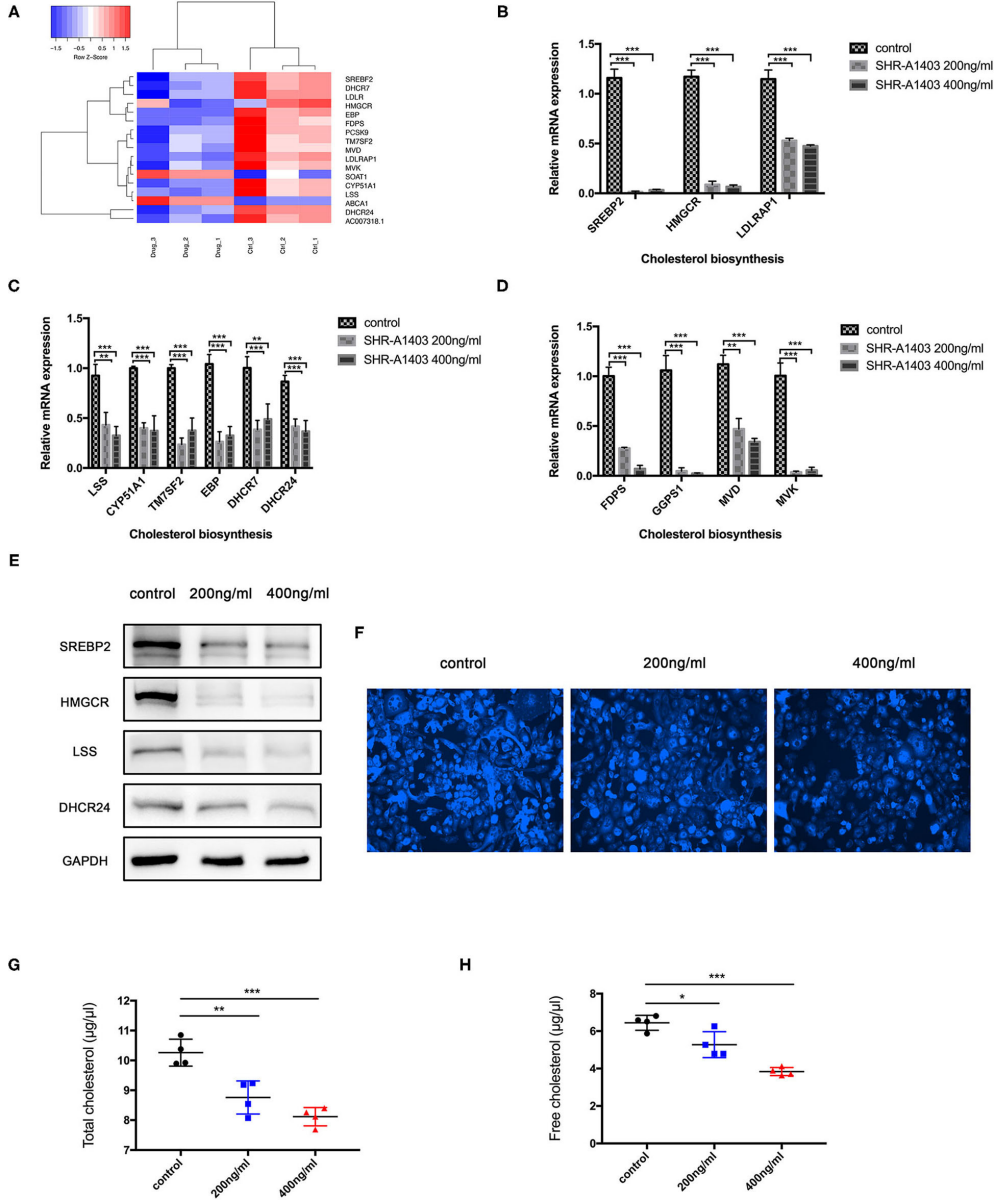
SHR-A1403 inhibits cholesterol biosynthesis in pancreatic cancer cells *in vitro*. **(A)** Heatmap shows the upregulated and downregulated genes in the cholesterol biosynthesis pathway in cells treated with SHR-A1403 compared to control-treated cells. **(B–D)** qRT-PCR analysis of key enzymes in the biosynthesis of cholesterol in PDAC cells treated with SHR-A1403. We examined enzymes involved upstream and downstream of biosynthesis, as well as those regulating intermediate metabolites. **(E)** Western blotting verified the expression of some key enzymes upstream and downstream of cholesterol biosynthesis in PDAC cells treated with SHR-A1403. **(F)** Fluorescence semi-quantitative detection of cholesterol content in Aspc-1 cells treated with different concentrations of SHR-A1403. **(G,H)** A cholesterol quantification kit was used to quantify the total cholesterol and free cholesterol content in Aspc-1 cells after treatment with different concentrations of SHR-A1403. *P < 0.05, **P < 0.01, ***P < 0.001. qRT-PCR, quantitative reverse transcription polymerase chain reaction; PDAC, pancreatic ductal adenocarcinoma.

Cluster analysis was performed on the differentially expressed genes of the cholesterol biosynthesis pathway to examine the expression and distribution of genes in the SHR-A1403-treated and control cells (Figure 4A). To test the hypothesis that SHR-A1403 inhibits cholesterol biosynthesis, we measured the mRNA and protein levels of key enzymes involved in cholesterol biosynthesis in cells treated with 200 and 400 ng/mL SHR-A1403. Consistent with the sequencing results, qRT-PCR showed that enzymes related to the mevalonate pathway and key enzymes downstream of lanosterol synthesis were significantly downregulated by SHR-A1403 (Figures 4B,C). Furthermore, key enzymes in the synthesis of important metabolites in the cholesterol biosynthesis pathway, including non-sterol isoprenoids, were also significantly downregulated (Figure 4D). We selected SREBP-2, HMGCR, LSS, and DHCR24, key enzymes in the regulation of cholesterol biosynthesis, for verification of this downregulation at the protein level. These enzymes were also significantly downregulated by SHR-A1403 at the protein level, which further confirmed the accuracy of our RNA analysis (Figure 4E).

To further verify these results, we used a semi-quantitative fluorescence method to detect the total cholesterol concentration in the cells after treatment. The cholesterol content in the cells treated with SHR-A1403 was significantly lower than that in control cells (Figure 4F). Next, we used a specific kit to detect the concentration of total cholesterol and free cholesterol in the cells. Both total cholesterol and free cholesterol were significantly reduced in cells treated with SHR-A1403 (Figures 4G,H). Together, these findings indicate that SHR-A1403 can significantly inhibit the entire cholesterol biosynthetic pathway in PDAC cells.

### SHR-A1403 Has Significant Anti-Tumour Activity in Xenograft Mouse Models of PDAC

**Figure 5:**
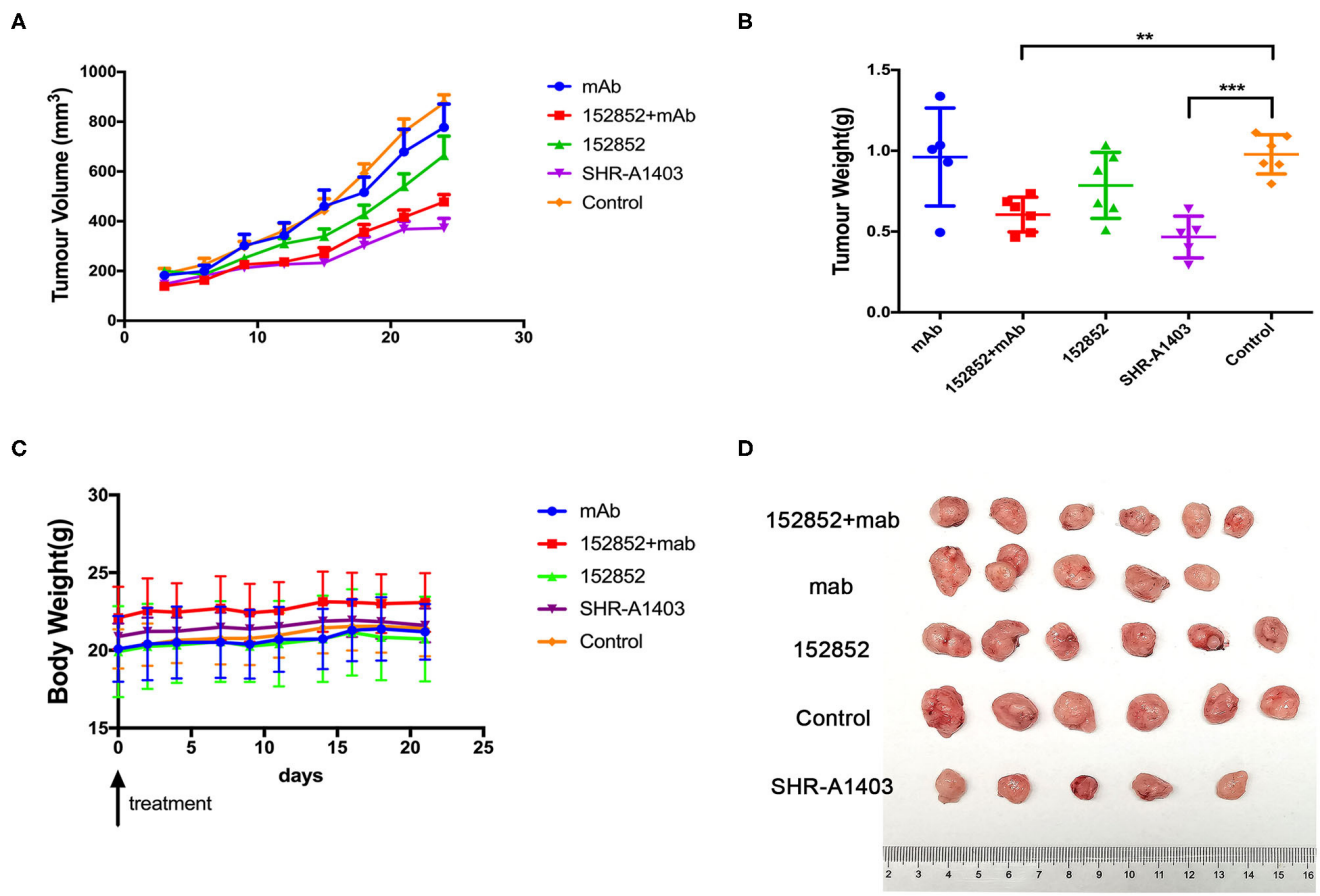
Anti-tumour activity *in vivo*. **(A,B)** The subcutaneous tumour xenograft models established with the Aspc-1 cell line were treated with vehicle, 10 mg/kg SHR-A1403, or 10 mg/kg SHR-A1403 mAb. The tumour volume and tumour weight were measured on the indicated days. **(C)** Body weight changes in the subcutaneous models in five treatment groups. **(D)** Mouse subcutaneous tumour images obtained after dissection. The data are shown as the mean ± SEM. **P < 0.01, ***P < 0.001. mAb, monoclonal antibody.

We wanted to further evaluate the anti-tumour efficacy of SHR-A1403 by examining its effect in xenograft mouse models. We injected human Aspc-1 cells, which have high c-MET, into BALB/c nude mice to establish a subcutaneous xenograft model. At the beginning of the treatment, nude mice weighed 20 to 25 g and ranged in age from 6 to 8 weeks. Drugs were administered by tail vein injection. We compared the therapeutic effect of SHR-A1403, SHR-A1403 mAb, free toxin, or a combination thereof. In this model, tumour-bearing mice were given SHR-A1403 10 mg/kg, SHR-A1403 mAb 10 mg/kg, SHR152852 0.11 mg/kg, or a combination of SHR-A1403 mAb+SHR152852 were injected intravenously into tumour-bearing mice. The SHR-A1403 dose was selected as the maximum safe dose of 10 mg/kg in mice based on previous experimental data (15, 16). The mAb and SHR152852 equivalent toxic doses were converted from this SHR-A1403 dose. We observed the treated animals for 21 days after treatment. As shown in Figure 5A, compared to the control group, the subcutaneous tumour volume of all treatment groups was reduced. The volume of subcutaneous tumours in the SHR-A1403 group was significantly reduced, and the volume of subcutaneous tumours in the SHR-152852 combined with mAb group was also relatively reduced, but overall, the effect was not as significant in the SHR-A1403 group. Regarding tumour weight, compared to the control group, the weight in the SHR-A1403 group decreased the most (*P* < 0.001), and the weight in the SHR-152852 combined mAb group also decreased. There was no significant difference between the other two treatment groups and the control group (Figure 5B). All groups tolerated the treatment well, and there was no significant difference in body weight before and after treatment (Figure 5C). Subcutaneous tumour images in the five groups are shown in Figure 5D.

## Discussion

In recent years, many studies have shown that MET plays a decisive role in the initiation, progression, and tumorigenicity of stem cells. The role of HGF/MET signalling in the pathogenesis of PDAC has also received increasing attention (17, 18). PDAC is characterised by a strong tumour stromal proliferative response, and the HGF/MET pathway is involved in the signal transmission interaction between tumour and stromal cells. Clinical studies have shown that elevated c-MET expression has prognostic value for many types of epithelial cancers (including pancreatic cancer) (19–21). Tumours overexpressing c-MET are associated with poor survival rates. As the c-MET expression is lower in corresponding normal tissues, such tumours are an ideal target for antibody-based biological therapy (22–24). In addition, c-MET in cancer cells is highly sensitive to antibody-induced internalisation, and the high efficiency of cytotoxic payloads to kill tumour cells requires antibody internalisation; therefore, c-MET therapy has inherent potential as a clinical anti-cancer option (25, 26).

ADCs are a type of biotherapeutic drug that can deliver cytotoxins (payloads) into cells using antibody targeting. They have shown good development prospects in basic research and clinical applications (12, 27). Considering the important role of MET in tumorigenesis and development and its limited progress in cancer treatment applications, the current development of ADCs targeting c-MET has been accelerated (28). SHR-A1403 is a new ADC that couples the humanised anti-MET mAb HTI-1066 and the auristatin analogue SHR152852 through a non-cleavable linker. HTI-1066 is highly specific to c-MET and blocks its dimerization and activation, which leads to a weakening of the intracellular signal cascade (16). SHR-A1403 has had good PK distribution and non-linear behaviour in preclinical studies (15). *In vitro* cytology experiments showed that SHR-A1403 can inhibit the proliferation of cancer cell lines and cause cell cycle arrest. *In vivo* experiments showed dose-dependent and long-lasting tumour growth-inhibitory effects in PDX models of hepatocellular carcinoma and gastric cancer cell line xenograft tumours. These results suggest that SHR-A1403 can effectively inhibit cancer cells that highly express c-MET both *in vivo* and *in vitro*. Previous mechanistic studies have shown that the anti-tumour efficacy of SHR-A1403 mainly depends on the significant inhibition of microtubule aggregation caused by internalised toxins and toxin metabolites and subsequent cell cycle arrest and is independent of signal transduction in downstream c-MET pathways (16). However, little has been published on the role of SHR-A1403 in PDAC. Our project proved the therapeutic prospect of SHR-A1403 in PDAC and explored a new mechanism for its anti-tumour effects.

Cholesterol is an essential component for cell survival and growth, and its metabolites perform a variety of biological functions that are necessary for cells to maintain normal activities. In the tumour microenvironment, the cholesterol biosynthesis pathway is reprogrammed, and various derivatives in the pathway play an important role in cancer progression and suppression of the immune response (29, 30). In pancreatic tumours, cholesterol can be regulated by signal transduction pathways involved in tumorigenesis and cancer progression to produce anabolic products, including non-sterol isoprenoids such as farnesyl pyrophosphate (FPP) and geranylgeranyl pyrophosphate (GGPP) (31). In this study, we demonstrated for the first time the therapeutic potential of SHR-A1403 in pancreatic tumours and discovered a new mechanism by which SHR-A1403 inhibits tumour cell proliferation, invasion, and migration. In contrast to previous studies showing that SHR-A1403 inhibits tumour growth through microtubule depolymerisation mediated by the intracellular release of SHR152852, we showed that, in PDAC, SHR-A1403 inhibits cholesterol biosynthesis, which is worthy of further study. The levels of key enzymes in the *de novo* cholesterol synthesis pathway and the LDL receptor-mediated absorption pathway were significantly downregulated in cells treated with SHR-A1403, and FPP and GGPP levels were decreased. Both FPP and GGPP are non-sterol isoprenoids that can activate a variety of oncoproteins, such as Ras, and are therefore important cholesterol metabolites that can promote tumorigenesis (32, 33). In addition, the final intermediate of cholesterol synthesis, lanosterol, can be further used as a substrate in the Bloch pathway, the Kandutsch-Russell pathway, and the hybrid pathway. Key enzymes involved in these pathways, such as DHCR24 and EBP, were also significantly downregulated (34).

**Figure 6:**
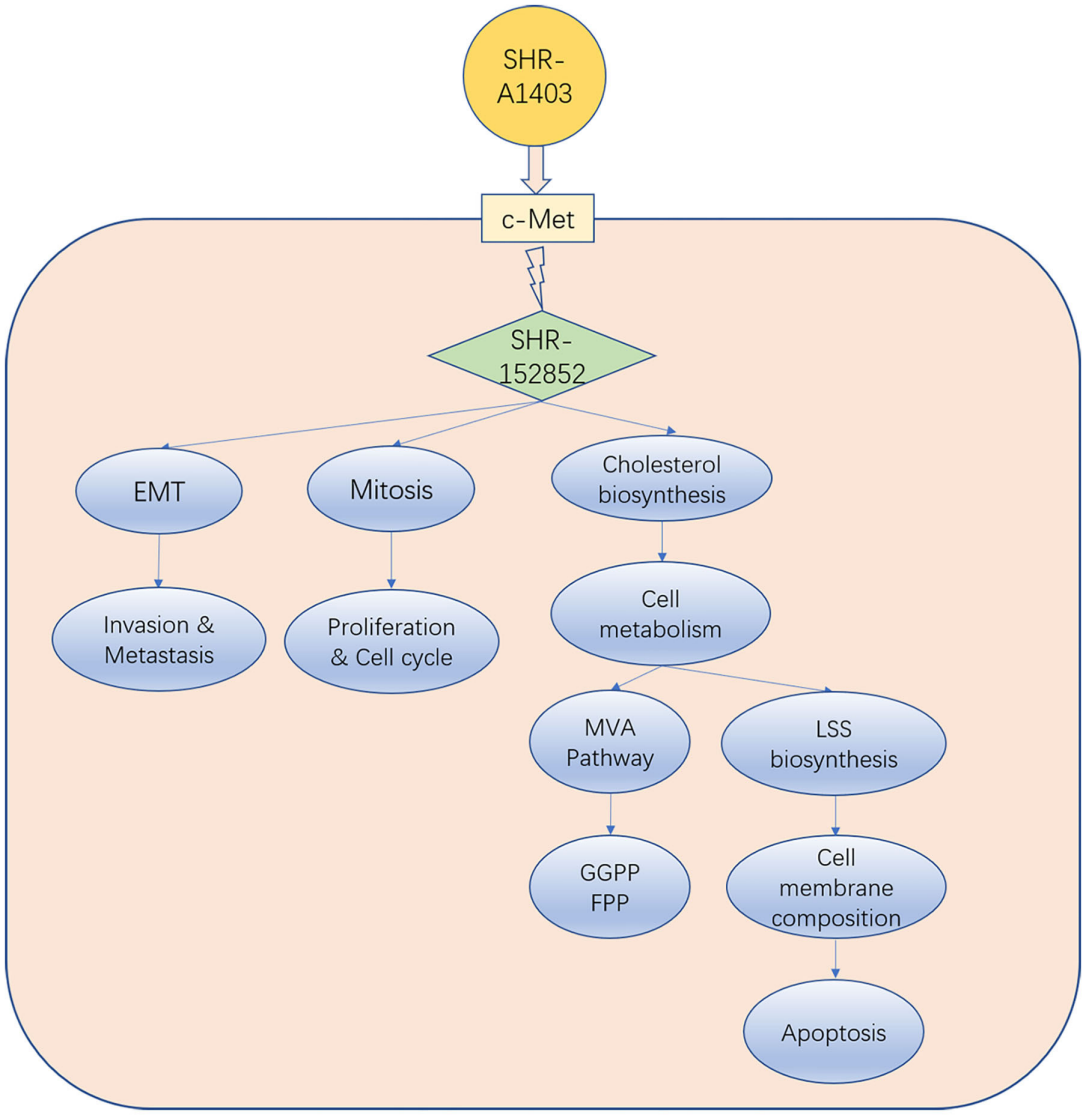
Model indicating how SHR-A1403 inhibits the progression of pancreatic ductal adenocarcinoma.

Unlike the current clinically available c-MET inhibitors and c-MET mAbs, which exert anti-tumour activity by inhibiting c-MET signal transduction, the new c-MET ADC SHR-A1403 exerts anti-tumour activity through the intracellular release of SHR152852. Our results and those of other studies suggest that SHR-A1403 inhibits PDAC growth through three mechanisms: (1) inhibition of cell proliferation and cell cycle by mediating microtubule depolymerisation; (2) inhibition of EMT to reduce the invasion and migration of pancreatic cancer cells; and (3) dysregulation of cellular metabolism by inhibiting the biosynthesis of cholesterol. Inhibition of the MVA pathway can reduce the production of intermediate metabolite non-steroidal isoprenoids (such as FPP and GGPP) and weaken Ras-related tumorigenesis, which may block pro-tumour downstream signal transduction. In addition, inhibition of LSS directly reduces downstream cholesterol synthesis, which blocks the synthesis of tumour cell membrane structure barriers and causes cell apoptosis. Therefore, these three pathways influence and promote each other, and blocking all of them simultaneously promotes the anti-tumour effect of SHR-A1403 in pancreatic cancer cells (Figure 6).

SHR-A1403 is thought to be a good candidate because of the low c-MET expression in normal tissues. However, to verify that there was no off-target toxicity of SHR-A1403 in targeting c-MET, c-MET was knocked down in a PDAC cell line with high c-MET expression, and cells were treated with SHR-A1403 or control. RNA was then collected and used for RNAseq. The differentially expressed genes and involved pathways were compared with those in wild-type cells treated with SHR-A1403 or control. There were fewer differentially expressed genes and involved pathways in the KD cells, which confirmed the weak off-target toxicity of SHR-A1403. The anti-tumour efficacy of SHR-A1403 was further confirmed in a PDAC xenogeneic model with high expression of c-MET. The tumours had a weak response to the naked antibody SHR-A1403 mAb and the small molecule toxin SH152852.

Other ADCs targeting c-MET are in development. ABBV-399, developed by Abbvie, has shown the fastest progress. ABBV-399 is composed of the c-MET-targeting antibody ABT-700 coupled to the toxin MMAE and uses a valine-glutamine acid cleavable linker. The DAR value of ABBV-399 is 3.1, which indicates non-fixed coupling (35, 36). However, the safety window of SHR152852 (the free toxin of SHR-A1403) is 64 times higher than that of MMAE, and the DAR value is close to 2. Moreover, because SHR-A1403 binds to free toxins through non-cleavable linkers, it can overcome potential defects seen in ABBV-399. In addition, SHR152852 has been proven to have relatively low cell permeability, further demonstrating its acceptable safety and providing an important reference for the controllable risks in human trials.

This study provides several key advances, as follows: We have verified the role of c-MET targeting ADC drugs in pancreatic cancer *in vivo* and *in vitro* for the first time. In addition, we explored the deep-level molecular mechanism through transcriptomics and found that SHR-A1403 could inhibit the cholesterol metabolism pathway, which is a simultaneous dual effect on metabolic pathways and molecular signal transduction pathways. This study could provide an early theoretical and practical basis for ADC drugs in the treatment of pancreatic cancer and lay the foundation and pioneering direction for the future clinical treatment of pancreatic cancer with new ADC drugs. However, our research has some limitations. First, compared with *in-situ* and PDX models, the biological characteristics and efficacy evaluation results of the subcutaneous tumour models are lower with clinical similarities; second, the research on the drug mechanism is still insufficient, and further investigation is needed to establish the mechanism of action; finally, the drug's efficacy in PDAC requires more preclinical experiments, such as ADC treatment combined with chemotherapy to determine the increase in efficacy.

In conclusion, SHR-A1403 is a new type of c-MET-targeting therapy. Our results suggest it has excellent potential to overcome the limitations of current treatments for pancreatic cancer, including currently available c-MET-targeting therapies. SHR-A1403 needs to be further evaluated to determine whether it is suited for pancreatic cancer therapy.

## Data Availability Statement

The datasets presented in this study can be found in online repositories. The names of the repository/repositories and accession number(s) can be found in the article/Supplementary Material.

## Ethics Statement

The studies involving human participants were reviewed and approved by relevant experiments involving human specimens were conducted in strict accordance with the Human Experiment Ethics Committee and the Declaration of Helsinki, 2013 revision. The patients/participants provided their written informed consent to participate in this study. The animal study was reviewed and approved by the ethics committee of Shanghai Ruijin Hospital (No. 17, 2019).

## Author Contributions

YJ, ZZ, SZ, and FL: experiments and manuscript development. CW and QZ: writing. HC, CP, XD, and BS: concept and supervision. All authors contributed to the article and approved the submitted version.

## Conflict of Interest

The authors declare that the research was conducted in the absence of any commercial or financial relationships that could be construed as a potential conflict of interest.

## Abbreviations

ADC: antibody-drug conjugate
c-MET: c-mesenchymal-epithelial transition factor
EMT: epithelial-mesenchymal transition
FPP: farnesyl pyrophosphate
GGPP: geranylgeranyl pyrophosphate
GTEX: The Genotype-Tissue Expression
HGF: hepatocyte growth factor
IHC: immunohistochemistry
IPA: ingenuity pathway analysis
KD: knockdown
mAb: monoclonal antibody
PDAC: pancreatic ductal adenocarcinoma
PDX: patient-derived xenografts
PI: propidium iodide
qRT-PCR: quantitative reverse transcription polymerase chain reaction
RNAseq: RNA sequencing
TCGA: The Cancer Genome Atlas.
