# Supplementary material for: A Novel c-MET-Targeting Antibody-Drug Conjugate for Pancreatic Cancer
Source: Front Oncol. 2021 Mar 17;11:634881. doi: 10.3389/fonc.2021.634881 (PMC8010262; doi:10.3389/fonc.2021.634881)
Supplement: Supplementary file 1 [file Table_1.DOCX]

PCR Primers

| GAPDH | FORWARD CAGGAGGCATTGCTGATGAT  REVERSE GAAGGCTGGGGCTCATTT |
| --- | --- |
| MET | FORWARD ATCAGGAGGTGTTTGGAAAGAT  REVERSE CGACTGTATGTCAGCAGTATGA |
| DHCR24 | FORWARD GAGTCATCATCCCACAAGTACG  REVERSE TAGAACAGGTCTGAGTTTTCGG |
| DHCR7 | FORWARD TGATTGACTTCTTCTGGAACGA  REVERSE TCATCTGCAGCGTGTAAAGATA |
| TM7SF2 | FORWARD GATATCACACATGACGGGTTTG  REVERSE CGGAAAGTGTTTTTCTGGGAA |
| LSS | FORWARD GAGAACGGCTCTGCGATGCTG  REVERSE GCTTGGTCTCATAGGTGGCGAAC |
| EBP | FORWARD AACTCTGGAAAGAGTATGCCAA  REVERSE AGACCACAAGCTGTAGAATGAA |
| CYP51A1 | FORWARD AGGCGATGGAGAAGGTGACAGG  REVERSE GTAGACCAGGCTGAGGGTGAAGG |
| HMGCR | FORWARD ATACAAGTATAGCTGGACGCAA  REVERSE CTGCATTTCAGGGAAATACTCG |
| SREBP2 | FORWARD AGCAGCAGCAGCAGCAATGG  REVERSE CGCCGAGGGAGAGAAGGAAGG |
| LDLRAP1 | FORWARD TTATCCTGACAGACAACCTCAC  REVERSE CATCTTGTCTGCTGTGCAATAG |
| FDPS | FORWARD ACCCAGAGATAGGAGATGCTAT  REVERSE GACTATCAGCATCCTGTTTCCT |
| GGPS1 | FORWARD GAAGACAACTCAAAACTCCGAC  REVERSE ATCTGGGTGATCAAGGGTTAAG |
| MVK | FORWARD ATGTTGTCAGAAGTCCTACTGG  REVERSE TCTCAAGTTCAAGGATACAGCC |
| MVD | FORWARD TCTCTTACCTCAATGCCATCTC  REVERSE CTTCAGAAACGTGTCTCCATTC |
