# Supplementary figures and images for: A Novel c-MET-Targeting Antibody-Drug Conjugate for Pancreatic Cancer
Source: Front Oncol. 2021 Mar 17;11:634881. doi: 10.3389/fonc.2021.634881 (PMC8010262; doi:10.3389/fonc.2021.634881)

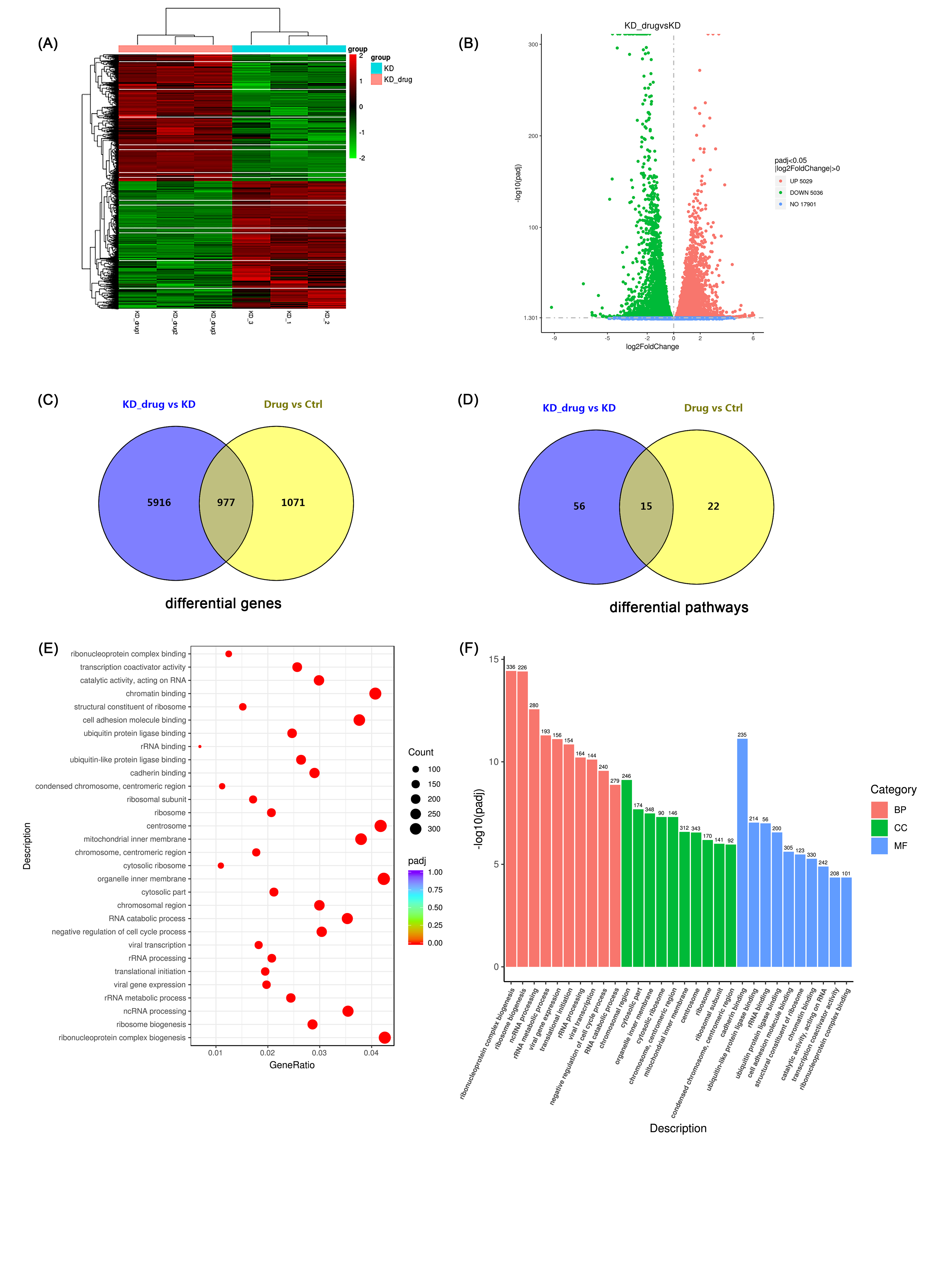

Supplement: Supplementary file 2 [file Image_1.TIF]
